# Auditory Contagious Yawning in Humans: An Investigation into Affiliation and Status Effects

**DOI:** 10.3389/fpsyg.2015.01735

**Published:** 2015-11-07

**Authors:** Jorg J. M. Massen, Allyson M. Church, Andrew C. Gallup

**Affiliations:** ^1^Department of Cognitive Biology, University of Vienna, Vienna, Austria; ^2^Department of Psychology, State University of New York at Oneonta, Oneonta, NY, USA; ^3^Psychology Program, Bard College, Annandale-on-Hudson, NY, USA

**Keywords:** emotional contagion, group vigilance, state matching, ingroup bias, auditory perception

## Abstract

While comparative research on contagious yawning has grown substantially in the past few years, both the interpersonal factors influencing this response and the sensory modalities involved in its activation in humans remain relatively unknown. Extending upon previous studies showing various in-group and status effects in non-human great apes, we performed an initial study to investigate how the political affiliation (Democrat vs. Republican) and status (high vs. low) of target stimuli influences auditory contagious yawning, as well as the urge to yawn, in humans. Self-report responses and a subset of video recordings were analyzed from 118 undergraduate students in the US following exposure to either breathing (control) or yawning (experimental) vocalizations paired with images of former US Presidents (high status) and their respective Cabinet Secretaries of Commerce (low status). The overall results validate the use of auditory stimuli to prompt yawn contagion, with greater response in the experimental than the control condition. There was also a negative effect of political status on self-reported yawning and the self-reported urge to yawn irrespective of the condition. In contrast, we found no evidence for a political affiliation bias in this response. These preliminary findings are discussed in terms of the existing comparative evidence, though we highlight limitations in the current investigation and we provide suggestions for future research in this area.

## Introduction

Accumulating research over the past decade has vastly improved our understanding of contagious yawning. Unlike spontaneous yawning, which is evolutionarily older and believed to be relatively widespread among vertebrates (Baenninger, 1987), contagious yawning is a more recently derived behavior present in relatively few highly social species (Gallup, 2011). In particular, contagious yawning has been reported in humans (e.g., Provine, 1986; Platek et al., 2003), some non-human primates including chimpanzees (e.g., Anderson et al., 2004; Campbell et al., 2009), bonobos (Demuru and Palagi, 2012; Palagi et al., 2014), and gelada baboons (Palagi et al., 2009); in wolves (Romero et al., 2014), in domesticated dogs in response to human yawns (Joly-Mascheroni et al., 2008; Madsen and Persson, 2012; Silva et al., 2012; Romero et al., 2013; but see Harr et al., 2009; O’Hara and Reeve, 2011; Buttner and Strasser, 2014), in a sub-line of high frequency yawning rats (Moyaho et al., 2015), and in budgerigars (Miller et al., 2012a; Gallup et al., 2015).

While spontaneous yawning appears to be triggered by deviations in brain thermal homeostasis (Gallup and Gallup, 2007, 2008; Shoup-Knox et al., 2010; Gallup and Eldakar, 2013; Massen et al., 2014; Eldakar et al., 2015), which could serve as a potential mechanism to promote cortical arousal (Baenninger, 1997) or state change (Provine, 1986; Liang et al., 2015), contagious yawning is elicited simply by sensing or thinking about the action (Provine, 2005). Consistent with this mode of response activation, it has been hypothesized that contagious yawning is rooted within a perception-action mechanism tied to basic forms of empathic processing (Preston and de Waal, 2002). A growing literature shows an indirect association between contagious yawning and empathy, both behaviorally (Platek et al., 2003; Palagi et al., 2009; Campbell and de Waal, 2011, 2014; Demuru and Palagi, 2012; Norscia and Palagi, 2011; de Waal, 2012; Romero et al., 2013, 2014; Silva et al., 2012; Rundle et al., 2015; but see Bartholomew and Cirulli, 2014) and neurologically (Platek et al., 2005; Arnott et al., 2009; Nahab et al., 2009; Cooper et al., 2012; Haker et al., 2013; but see Schurmann et al., 2005; Gallup and Church, 2015). Studies investigating the developmental onset of contagious yawning in children also generally support this view (Anderson and Meno, 2003; Millen and Anderson, 2010; Hoogenhout et al., 2013), since contagious yawning develops in parallel with empathy related capacities (e.g., Perner and Lang, 1999). Initial reports on the absence of contagious yawning in children with autism spectrum disorder also supported this connection to empathy (Senju et al., 2007; Giganti and Ziello, 2009; Helt et al., 2010), but subsequent research shows that this result may be a consequence of the reduced tendency for these individuals to spontaneously attend to others’ faces (Senju et al., 2009; Usui et al., 2013). Consequently, the link between contagious yawning and empathy remains debated (see Yoon and Tennie, 2010). Nonetheless, while contagious yawning may serve as a useful marker for social-psychological functioning, future research into this area is warranted.

While the adaptive value of contagious yawning remains largely unclear, some recent experimental research suggests a role in promoting group coordination and/or vigilance (Miller et al., 2012b). Indirect support for this view also comes from a growing number of behavioral reports investigating contagious yawning in relation to group affiliation or social closeness/bonding. In particular, an influential study on chimpanzees demonstrated evidence for an in-group bias for contagious yawning (Campbell and de Waal, 2011). In particular, captive chimpanzees shown video stimuli of other chimpanzees yawning showed contagion to in-group members but not unfamiliar conspecifics. However, a similar familiarity bias has not been demonstrated for chimpanzees viewing human yawns (Madsen et al., 2013; Campbell and de Waal, 2014), and at least one study provided evidence that relationship quality among chimpanzees within a single group did not predict yawn contagion (Massen et al., 2012). Nonetheless, similar in-group findings involving a measure of social closeness have also been demonstrated in humans (Norscia and Palagi, 2011), gelada baboons (Palagi et al., 2009), bonobos (Demuru and Palagi, 2012; Palagi et al., 2014), and wolves (Romero et al., 2014), with mixed support for domesticated dogs to catch familiar human yawns (O’Hara and Reeve, 2011; Silva et al., 2012; Madsen and Persson, 2012; Romero et al., 2013). For example, naturalistic observations on humans have revealed contagious yawning to be of significantly higher frequency when witnessing kin and friends yawn, in comparison to acquaintances and strangers (Norscia and Palagi, 2011). In great apes, the status or dominance of the target individual (i.e., the yawner) also seems to influence the susceptibility for others to catch this behavior (Demuru and Palagi, 2012; Massen et al., 2012). For example, chimpanzees and bonobos are more likely to yawn in response to witnessing yawns from conspecifics of the dominant sex (i.e., males in chimpanzees, females in bonobos). It remains unclear, however, whether the yawns of dominant chimpanzees and bonobos are more contagious or that individuals just pay more attention to dominants, which is consistent with the view that monitoring high status or dominant individuals will provide important information regarding changes in reproductive opportunities, group state or vigilance (Chance, 1967; Keverne et al., 1978; McNelis and Boatright-Horowitz, 1998; Shepherd et al., 2006; Overduin-de Vries et al., 2012). Similarly, one could argue that individuals pay more attention to their friends and/or in-group members, explaining the related differences in contagiousness of yawns. Unfortunately, however, aside from that mentioned above, little is known about such in-group and status effects on contagious yawning in humans.

The current research was designed to build from this existing comparative literature by investigating how some of these interpersonal variables influence contagious yawning in humans. Specifically, based on a study showing an effect of in-group voter biases of reflexive gaze-following to politicians (Liuzza et al., 2011), we tested whether a similar effect would be present when activating contagious yawning. Since humans have developed large-scale political behavior and evolved in relatively large societies, political identity or affiliation may provide salient in-group/out-group cues that modulate other unconscious social responses. Previous research has shown that neural activity differs markedly when viewing images of US Presidential candidates of the same vs. opposing political party (Kaplan et al., 2007). For example, viewing opposing-party candidates activated areas of negative emotion (e.g., insula, anterior temporal cortex, anterior cingulate cortex, and dorsolateral prefrontal cortex) while, at least for Democrats, viewing same-party candidates enhanced activation in areas associated with positive emotion and empathy (i.e., the medial prefrontal cortex). Furthermore, distinct patterns of neural activity have been shown to occur within brain regions associated with theory of mind when participants were asked to take the perspective of ingroup vs. outgroup political candidates (Falk et al., 2012). In particular, the posterior cingulate cortex becomes more active during perspective taking of an ingroup political candidate, while the bilateral temporoparietal junction was more active when considering an outgroup candidate’s political views.

While it has been confirmed that both seeing and thinking about yawns can trigger contagious yawning in humans (i.e., Provine, 1986), it has been reported that hearing yawns can produce the same effect (Provine, 2005). One study has used audio recordings of yawns to assess the self-reported urge to yawn in humans during fMRI scans (Arnott et al., 2009), but to date there is no evidence showing that auditory cues presented in isolation can elicit yawn contagion in humans. Similarly, comparative studies have primarily used visual stimuli for tests of contagious yawning in non-human animals. Currently only two studies have addressed whether auditory cues alone can trigger this response. Experimental research has provided evidence for cross-species (human to canine) auditory contagious yawning in domesticated dogs (Silva et al., 2012), and an observational study has shown an increase in yawning frequency among gelada baboons when in the presence of auditory cues from nearby conspecifics (Palagi et al., 2009). In this initial investigation we manipulated the political affiliation (Democrat vs. Republican) and status (former US Presidents vs. Cabinet members) of our target stimuli, which were paired with either breathing or yawning sounds. This is the first study to test the effectiveness of this priming stimulus in actually eliciting yawns in humans. Consistent with previous comparative research on empathy and perspective taking, social affiliation and status, and auditory contagion, we hypothesized that participants would yawn significantly more when hearing yawning sounds paired with images of same-party, high status political figures.

## Materials and Methods

### Participants

This study included 118 undergraduate psychology students (36 male; 82 female) from two separate colleges in Upstate New York in Spring 2013 and Fall 2014. Participants ranged in age from 18 to 24 (mean age: 18.76; SD = 1.20), and thus were either children or adolescents during the presidential terms of our stimulus targets. Each individual gave verbal and written consent to participate in this study. The individual Institutional Review Boards at Bard College and State University of New York at Oneonta approved this research and the consent procedure (respective reference numbers: OCT12GAL1; 2014-94). All participants were given course credit for participation through an online portal, and responses to questionnaires were held anonymous.

### Procedure

Participants were seated in a testing room in front of a desk with a computer screen and given instructions from a research assistant regarding the procedures of the experiment. First, they completed a written demographic survey including questions regarding their age, sex, ethnicity, political affiliation, and political engagement. Next, participants were given instructions presented to them on a PowerPoint presentation. The instructions read as follows:

Slide 1: “*You have now completed the first part of the experiment. In the next portion of this experiment, you will be shown a series of images of politicians with varying power and affiliations. There will be four politicians, each with three pictures apiece, for a total of 12 images.*”

Slide 2: “*The images of each politician will be paired with auditory clips taken from that individual (breathing sounds). You will be asked to view the series of three images for each person separately while listening carefully to the audio clips. Important: Be sure to listen to all of the audio clips before moving to the next slide. These will last close to one minute on each slide.*”

Slide 3: “*At the end of each section, you will be asked to fill out some information about the person you were just viewing before moving to the next. Do you have any questions? The experimenter will now be leaving the room and will be waiting outside. Please be sure you do not have any questions before they leave.*”

At this point participants were fitted with headphones linked to the computer. Although the instructions indicated they would be listening to breathing sounds only, the participants were actually randomly assigned to either the control (breathing sounds) or experimental condition (yawning sounds). With permissions from the authors, we used the same auditory stimuli from a previous study investigating changes in localized brain activity in response to hearing yawns (Arnott et al., 2009). Since previous research suggests that participants are less likely to yawn when they are being observed (Baenninger, 1987; Baenninger and Greco, 1991), the researcher left the room at this stage of the experiment after all questions had been answered. Participants then began to perform the semi-automated task alone in the testing room while the research assistant waited outside.

The political stimuli were presented on individual PowerPoint slides as pictures of former US Presidents William Jefferson “Bill” Clinton and George Walker Bush, and their former Secretaries of Commerce William Daley (serving under Clinton) and Carlos Gutierrez (serving under Bush). There were 12 pictures in total, representing three slides for each politician. All pictures were in color, roughly 7.5 × 10 cm in size, and placed on a white background. The pictures were carefully selected from the Internet for purposes of uniformity in the appearance of the individual (i.e., all images included front-view shots of the politicians’ head and shoulders where they were smiling and wearing a suit and tie). Therefore, we used a 2 × 2 design including two former US presidents and two lower status politicians, and similarly two Democrats and two Republicans. Presidents Clinton and Bush were chosen because they represent salient and high status members of the Democratic and Republican political parties (i.e., HS Dem and HS Rep), while secretaries Daley and Gutierrez were chosen because they represent relatively less familiar and lower status political figures (i.e., LS Dem and LS Rep). While dynamic scenes of these politicians would have likely elicited a higher neuro-physiological response (e.g., Trautmann-Lengsfeld et al., 2013), archived footage of these individuals would have been highly variable in terms of behavior and context of the scene. The images we used were chosen due to our ability to standardize the presentation for each politician (size, background, personal attire, facial expression, etc.). Thus, in all cases the visual representation was highly consistent.

At the top of each stimulus slide appropriate descriptions were also given above the image to explicitly represent both political and status features. For example, above the images of President Clinton it read “Democrat of high status,” while above former Secretary Gutierrez it read “Republican of low status.” Previous research has shown that comparable status labeling procedures alter perception and responses to stimulus targets (e.g., de Kwaadsteniet and van Dijk, 2010; Lount and Pettit, 2012). Each participant viewed three consecutive images of each political figure before moving on to the next. The order of presentation between subjects was partially counterbalanced for political party (Dem vs. Rep) and status (HS vs. LS), and randomly assigned to participants. The presentation order included the following four separate trials: (1) HS Rep; LS Rep; HS Dem; LS Dem, (2) LS Rep; HS Rep; LS Dem; HS Dem, (3) HS Dem; LS Dem; HS Rep; LS Rep, and (4) LS Dem; HS Dem; LS Rep; HS Rep.

Each image was paired with 10 successive audio clips (ranging from 4 to 9 s) that played automatically at the beginning of the slide, depicting either breathing or yawn sounds generated by Arnott et al. (2009). The different sounds were randomly assigned to the separate trials of different participants (control/breathing: *N* = 55; experimental/yawning: *N* = 63). As discussed above, the participants were told that these “breathing” sounds were from the politician on the screen. At the completion of the sequence of auditory clips, the participants then manually clicked to the next slide. After viewing three consecutive slides with the same politician, a subsequent slide provided a brief pause between political figures, serving two purposes. First, given the relatively slow latency for yawn contagion in comparison to other reflexive and involuntary contagious responses, it allowed for instances of delayed contagious yawning to be contained within the stimulus presentation that triggered them. Second, it prompted participants to fill out a short written survey consisting of three questions pertaining to the political figure they were just exposed to: (1) Did you recognize this person, and if so who is it? (2) How much do you personally like or affiliate with this person? (7-point Likert scale), and (3) How much do you agree with the political views of this person? (7-point Likert scale). These questions were included to control for personal affiliations or feelings toward these past politicians, as well as determine familiarity with these individuals. Not a single participant identified either of the former Secretaries, while 90.7 and 83.1% of participants accurately identified Presidents Bush and Clinton, respectively. Thus, feelings of likeness and agreement were restricted to the former Presidents, and thus not included in the overall analyses.

At the end of the PowerPoint presentation when all four political figures had been viewed, participants responded to a post-experiment written survey on whether they yawned during the experiment or had the urge to do so at any time. If so, they were asked to identify which section their yawn(s) and urge(s) occurred by placing respective checks next to the following statements: “When viewing images of the high-status Democrat,” “When viewing images of the low-status Democrat,” “When viewing images of the high-status Republican,” “When viewing images of the low-status Republican.” Participants could then be categorized into yawners or non-yawners, with a total number of yawning trials ranging from 0 to 4. Following the completion of the experiment, participants were debriefed and asked not to discuss the rationale of the experiment with fellow students.

A total of 40 participants (33.9%) were recorded with a webcam positioned on the computer monitor to further validate their self-report measure (cf. Greco and Baenninger, 1989). For this sample two independent observers scored the recordings to confirm all yawns. Since the webcam was facing the participants, and the length of time spent in the experiment was not standardized, the total recording time was divided by four and any yawns were then designated according to the quartile of the video in which they occurred, and subsequently matched to the appropriate political figure for that trial order. The inter-rater reliability of self-report vs. actual yawns was calculated using Cohen’s Kappa. We found substantial agreement between actual yawns and self-reported yawning during the experiment (*k* = 0.67), confirming previous research (Greco and Baenninger, 1989; Gallup and Church, 2015). In terms of the assignment of specific yawns to political figures, the agreement (80%) was only fair (*k* = 0.22), suggesting that the video quartiles did not necessarily correspond to the appropriate trial order or that there might be a bias in the self-report.

### Analyses

Experimental testing times were scheduled from 09:45 to 18:30 h, with the total procedure taking roughly 20 min. The testing time was noted on the paper survey for all but three participants. We found no differences in testing time for yawners (*N* = 48) vs. non-yawners (*N* = 67; *t*_113_ = 0.080, *p* = 0.937), and there were no differences in yawning rates as function of political presentation order [yawners vs. non-yawners: *χ*^2^_(3)_ = 0.649, *p* = 0.885; total yawns: *F*_3,117_ = 0.159, *p* = 0.924]. Data regarding the urge to yawn provided very similar results (*p*s = ns). Lastly, there was no difference in experimental testing times between the control (breathing) and experimental (yawning) conditions (*t*_113_ = –0.712, *p* = 0.478). Thus, unless specified the following analysis included all participants.

It should be noted that our sample showed a liberal bias in their political affiliation, including 75 self-reported Democrats, 13 Republicans, and 30 remaining participants identifying themselves as either Independent or other (i.e., Libertarian, Green Party, etc.). We used separate general linear mixed models (GLMM) with a binomial distribution (logit link function) to assess the influence of multiple variables on the likelihood that a participant would respond with a yawn or the urge to yawn, respectively. Age was entered into these GLMM’s as a fixed covariate, since small differences in age could represent substantial differences in participants’ age when politicians were still in office. Sex and political affiliation of the participant (we tested both the actual affiliation and a categorization of Democrat vs. all other parties), as well as the interaction between the participants’ political affiliation and their political engagement, the political affiliation of the stimulus (Democrat vs. Republican), the status of the political figure (high vs. low), whether the stimulus belonged to the political party the participant affiliated with (in- vs. out-group), and condition (breathe vs. yawn) were entered as fixed factors. Since Secretary Gutierrez represented the only non-white political figure, we also explored ingroup and outgroup effects based on ethnicity but no significant findings emerged. In addition, we included all two-way interactions of condition with all other variables/interactions. Moreover, as we used a repeated measure design, subject ID and the order of stimuli were entered as random factors. To achieve the best models we used a backward stepwise approach, and our model choices were based on comparisons of the Corrected Akaike Information Criteria (CAIC). For reasons of clarity, here we present only the best fitting models. *Post hoc* analyses included Fisher’s exact or McNemar’s tests (with Yates’s correction for continuity) depending on whether the tests were between or within participants, respectively. All analyses were performed in IBM SPSS Statistics v. 20 for Mac OS, with α set to 0.05.

## Results

**FIGURE 1:**
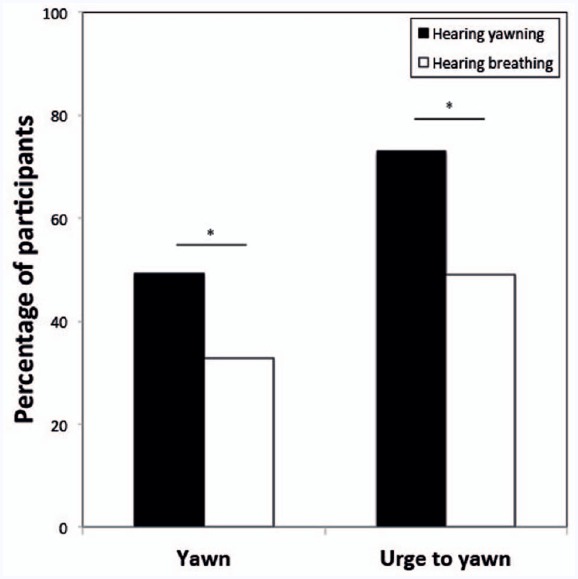
**Proportion of participants that responded with a yawn or had the urge to yawn while/after hearing either yawns (experimental condition, black bars) or breaths (control, white bars).** *p < 0.05.

**FIGURE 2:**
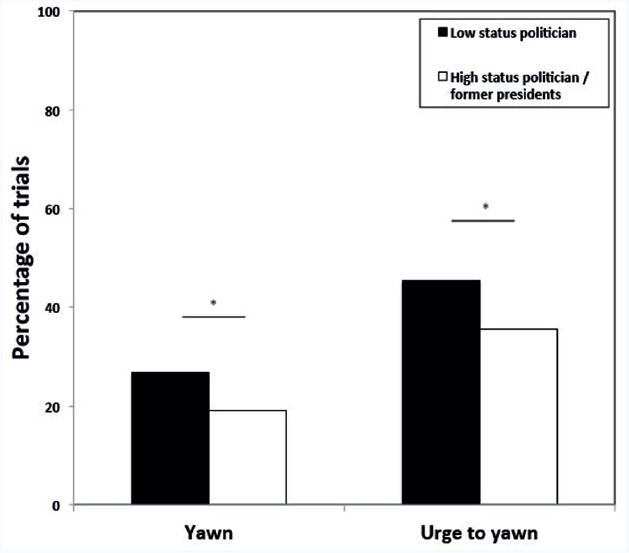
**Proportion of trials showing pictures of low status politicians (black bars) or high status politicians/former presidents (white bars) in which participants yawned or had the urge to yawn.** *p < 0.05.

The best fitting GLMM revealed that the likelihood of a participant to respond with a yawn to the political stimuli was significantly influenced by the type of auditory cue (breathe vs. yawn); i.e., significantly more participants reported yawning when listening to vocalized yawns compared to repeated breaths [31 out of 63 vs. 18 out of 55; β = 1.25 ± SE 0.40, *F*(1,469) = 9.67, *p* = 0.002; Figure 1]. In addition, the model revealed a significant effect of political status; i.e., significantly more participants reported yawning when viewing low status political figures compared to high status politicians [β = 0.59 ± SE 0.26, *F*(1,469) = 5.09, *p* = 0.025; Figure 2]. No other variables were significant.

When we tested the effect of the same variables on the urge to yawn, the best fitting model again revealed a significant effect of the auditory stimulus [breathe vs. yawn; β** = 1.44 ± SE 0.41, *F*(1,469) = 12.67, *p* < 0.001; Figure 1], as well as a main effect of political status [β = 0.61 ± SE 0.24, *F*(1,469) = 6.56, *p* = 0.010; Figure 2]. Again no other variables were significant.

Finally, to validate the reported results, we analyzed the data from the video recordings and compared the distributions of individuals that yawned based on condition and political status with the corresponding distributions of the self-reported data. We found no difference regarding the distribution of individuals that yawned in the experimental vs. the control condition between self-reported and actual video data (*χ*^2^: 0.066, df = 1, *p* = 0.797). In contrast, the distribution of individuals that yawned while seeing high-status vs. low-status political figures tended to differ between self-reported and actual video data, albeit not significantly (*χ*^2^: 2.783, df = 1, *p* = 0.095).

## Discussion

These findings add to our understanding of the sensory, perceptual and interpersonal factors contributing to (contagious) yawning in humans. The current study is the only one to our knowledge to show auditory contagious yawning in humans. This finding is consistent with an earlier study reporting that humans’ urge to yawn increases after having heard someone yawn (Arnott et al., 2009). Moreover, this finding is in line with a previous report on domesticated dogs demonstrating that hearing a yawn, albeit heterospecific (i.e., human), elicits contagious yawning in this species (Silva et al., 2012). In addition, there are observational indications that gelada baboons increase their yawning in the presence of yawning vocalizations of nearby conspecifics (Palagi et al., 2009). The current findings suggest auditory contagious yawning may also be a preserved mechanism present in mammalian species that show yawn contagion and not just restricted to those that are particularly well adapted with regard to hearing; e.g., dogs.

Our results also show that an auditory yawn stimulus is equally effective as visual stimuli for priming contagious yawning in humans, since 49.2% of the participants that heard a yawn reported a yawn in comparison to roughly 40–55% who yawn after seeing pictures or videos of people yawning (e.g., Provine, 1986; Platek et al., 2003; Gallup and Gallup, 2007; Massen et al., 2014). In addition, although it is possible that the participants suspected that the audio clips were not from the politicians themselves, this stimulus proved to be a good tool for priming contagious yawning with regard to particular visual stimuli, as we found several patterns in the reported contagiousness of yawning in response to hearing someone yawn that could only be attributed to the corresponding visual stimuli. Therefore, this methodology could be preferred when, as in the current study, visual yawning stimuli are highly variable and/or difficult to acquire.

Aside from auditory signal, our results demonstrate that the self-reported response to yawn was influenced by the status of the politicians in the pictures (see below for extended discussion of methodological limitations). We found that regardless of the auditory priming stimulus, be it yawning or breathing, participants reported yawning more when seeing a low status political figure than a high status political figure. This contrasts with findings on bonobos and chimpanzees, where yawns of high status/ranking individuals (females among bonobos, males among chimpanzees) were more contagious than those of low ranking individuals (Demuru and Palagi, 2012; Massen et al., 2012). For apes this effect could be a result of differences in attention since the monitoring of dominant animals might provide several advantages regarding changes in reproductive opportunities, group state or vigilance (Chance, 1967; Keverne et al., 1978; McNelis and Boatright-Horowitz, 1998; Shepherd et al., 2006; Overduin-de Vries et al., 2012). Our participants were specifically requested to pay equal attention to all stimulus images, and thus these results possibly suggest a discontinuity between apes and humans in the evolution of yawning in response to, or simply in the presence of, individuals of different status. This discontinuity may be a cultural effect, portraying that for humans yawning is often viewed as a sign of boredom or disrespect in the presence of others (Schiller, 2002), which may inhibit yawning in general when viewing individuals of extremely high status and reputation like the former presidents in our study. Such an inhibition hypothesis, however, would predict that the urge to yawn would be equal for both high and low status politicians, though that it would only be inhibited in case of the high status former presidents. This interpretation is supported by recent research demonstrating that administration of intranasal oxytocin produces a significant discrepancy between the self-reported urge to yawn and actual yawning rates of human participants, suggesting that individuals may consciously inhibit this response (Gallup and Church, 2015). Conversely, we found a similar status effect with regard to the urge to yawn. That said, the reduced self-reported yawning (and urge to yawn) in response to past US presidents might not generalize across other high status target stimuli. In fact, there may be a similar high status copying bias as seen in other great apes when using target individuals of varying social dominance/rank that are within the participants’ social network, such as among friends, coworkers or different social organizations.

Unlike previous research showing an in-group political bias for gaze-following (Liuzza et al., 2011), we found no evidence for similar results of self-reported contagious yawning. Multiple factors could explain this null effect. Most notably, our sample was rather homogeneous in their political views, with 63.6% identifying as Democrats and only 11.0% identifying as a Republican. Thus, future research could aim to draw from a larger and more representative population to test these effects. However, we found no evidence for an in-group bias among the Democrats to yawn more in response to President Clinton or former Secretary Daley. It should be noted here that we ran models with the actual political affiliation (which party) of our subjects as well as with a categorization of Democrats vs. all other parties, though the results were the same and neither variable was significant in the best fitting models. Since none of the political figures used as our target stimuli are still in office, and were so only when the participant sample was quite young, it also remains possible that using contemporary politicians or older participants would produce a greater in-group/out-group affiliation. Alternatively, despite differences in neural activity associated with viewing images or taking the perspective of politicians from congruent or opposing parties (Kaplan et al., 2007; Falk et al., 2012), political affiliation and/or political in-group affiliation may simply not be a strong enough cue to affect contagious yawning.

Although our results provide effects of unique interpersonal factors influencing yawning in humans, there are additional limitations to acknowledge within this preliminary study. In particular, the current study only utilized a single exemplar for each of the four stimulus conditions (HS Dem, LS Dem, HS Rep, LS Rep), and thus further investigation is needed in order to replicate and clarify these results. Furthermore, the self-report nature of our data is suboptimal. In order to keep the goals of the experiment implicit participants were asked to self-report on their yawning behavior at the end of the task, but this may have increased the chances of measurement error. The inclusion of the subset of video recorded participants confirmed the validity of overall self-report yawning behavior, and showed a similar distribution of individuals that yawned in respect to the auditory treatment. However, validation of recall of the specific political stimuli was only fair, and the distribution of individuals that yawned during exposure to high and low status political figures tended to differ between video and self-report data. Since the images of the politicians were explicitly labeled for status, it is possible that expectancy bias contributed to participants assigning fewer yawns in the presence of high status figures. Therefore, bias in self-reporting may have contributed to the status effect. Nonetheless, subjective responses in the current study provide understanding of human perception and may reveal important factors contributing to the social stigma associated with yawning in the presence of others. Future research could be conducted to improve upon methodological and sampling limitations and further explore the interplay between perceived and actual inhibition of behavioral contagion as it relates to social context.

In conclusion, the results of this investigation validate the use of auditory stimuli to prompt yawn contagion and provide evidence for an effect of target status influencing the self-reported nature of this response in humans. Despite the limitations in our sample and design, these initial findings broaden the existing literature and provide a clear framework for pursuing further research in this area.

### Conflict of Interest Statement

The authors declare that the research was conducted in the absence of any commercial or financial relationships that could be construed as a potential conflict of interest.

## References

[B1] Anderson J. R. Meno P. (2003). Psychological influences on yawning in children. Curr. Psychol. Lett. 11.

[B2] Anderson J. R. Myowa-Yamakoshi M. Matsuzawa T. (2004). Contagious yawning in chimpanzees. Proc. R. Soc. B Biol. Sci. 271, S468–S470. 10.1098/rsbl.2004.0224 PMC1810104 15801606

[B3] Arnott S. R. Singhal A. Goodale M. A. (2009). An investigation of auditory contagious yawning. Cogn. Affect. Behav. Neurosci. 9, 335–342. 10.3758/CABN.9.3.335 19679768

[B4] Baenninger R. (1987). Some comparative aspects of yawning in *Betta splendens*, *Homo sapiens*, *Panthera leo*, and *Papio sphinx*. J. Comp. Psychol. 101, 349–354. 10.1037/0735-7036.101.4.349

[B5] Baenninger R. (1997). On yawning and its functions. Psychon. Bull. Rev. 4, 198–207. 10.3758/BF03209394 21331826

[B6] Baenninger R. Greco M. (1991). Some antecedents and consequences of yawning. Psychol. Rec. 41, 453–460.

[B7] Bartholomew A. J. Cirulli E. T. (2014). Individual variation in contagious yawning susceptibility is highly stable and largely unexplained by empathy or other known factors. PLoS ONE 9:e91773. 10.1371/journal.pone.0091773 24632594 PMC3954725

[B8] Buttner A. P. Strasser R. (2014). Contagious yawning, social cognition, and arousal: an investigation of the processes underlying shelter dogs’ responses to human yawns. Anim. Cogn. 17, 95–104. 10.1007/s10071-013-0641-z 23670215

[B9] Campbell M. W. Carter J. D. Proctor D. Eisenberg M. L. de Waal F. B. M. (2009). Computer animations stimulate contagious yawning in chimpanzees. Proc. R. Soc. B Biol. Sci. 276, 4255–4259. 10.1098/rspb.2009.1087 19740888 PMC2821339

[B10] Campbell M. W. de Waal F. B. M. (2011). Ingroup-outgroup bias in contagious yawning by chimpanzees supports link to empathy. PLoS ONE 6:e18283. 10.1371/journal.pone.0018283 21494669 PMC3071812

[B11] Campbell M. W. de Waal F. B. M. (2014). Chimpanzees empathize with group mates and humans, but not with baboons or unfamiliar chimpanzees. Proc. R. Soc. B Biol. Sci. 281, 20140013. 10.1098/rspb.2014.0013 24619445 PMC3973272

[B12] Chance M. R. A. (1967). Attention structure as the basis of primate rank orders. Man 2, 503–518. 10.2307/2799336

[B13] Cooper N. R. Puzzo I. Pawley A. D. Bowes-Mulligan R. A. Kirkpatrick E. V. Antoniou P. A. (2012). Bridging a yawning chasm: EEG investigations into the debate concerning the role of the human mirror neuron system in contagious yawning. Cogn. Affect. Behav. Neurosci. 12, 393–405. 10.3758/s13415-011-0081-7 22198677

[B14] Demuru E. Palagi E. (2012). In bonobos yawn contagion is higher among kin and friends. PLoS ONE 7:e49613. 10.1371/journal.pone.0049613 23166729 PMC3498209

[B15] de Kwaadsteniet E. W. van Dijk E. (2010). Social status as a cue for tacit coordination. J. Exp. Soc. Psychol. 46, 515–524. 10.1016/j.jesp.2010.01.005

[B16] de Waal F. B. M. (2012). “Empathy in primates and other mammals,” in Empathy From Bench to Bedside, ed. Decety J. (Cambridge: MIT Press), 87–106.

[B17] Eldakar O. T. Dauzonne M. Prilutzkaya Y. Garcia D. Thadal C. Gallup A. C. (2015). Temperature-dependent variation in self-reported contagious yawning. Adapt. Hum. Behav. Physiol. 1, 460–466. 10.1007/s40750-015-0024-6

[B18] Falk E. B. Spunt R. P. Lieberman M. D. (2012). Ascribing beliefs to ingroup and outgroup political candidates: neural correlates of perspective-taking, issue importance and days until the election. Philos. Trans. R. Soc. Lond. B Biol. Sci. 367, 731–743. 10.1098/rstb.2011.0302 22271788 PMC3260850

[B19] Gallup A. C. (2011). Why do we yawn? Primitive versus derived features. Neurosci. Biobehav. Rev. 35, 765–769. 10.1016/j.neubiorev.2010.09.009 20883719

[B20] Gallup A. C. Gallup G. G. Jr. (2007). Yawning as a brain cooling mechanism: nasal breathing and forehead cooling diminish the incidence of contagious yawning. Evol. Psychol. 5, 92–101. 10.1177/147470490700500109

[B21] Gallup A. C. Eldakar O. T. (2013). The thermoregulatory theory of yawning: what we know from 5 years of research. Front. Neurosci. 6:188. 10.3389/fnins.2012.00188 23293583 PMC3534187

[B22] Gallup A. C. Gallup G. G. Jr (2008). Yawning and thermoregulation. Phys. Behav. 95, 10–16. 10.1016/j.physbeh.2008.05.003 18550130

[B23] Gallup A. C. Church A. M. (2015). The effects of intranasal oxytocin on contagious yawning. Neurosci. Lett. 607, 13–16. 10.1016/j.neulet.2015.09.007 26375928

[B24] Gallup A. C. Swartwood L. Militello J. Sackett S. (2015). Experimental evidence for contagious yawning in budgerigars (*Melopsittacus undulatus*). Anim. Cogn. 18, 1051–1058. 10.1007/s10071-015-0873-1 26012708

[B25] Giganti F. Ziello E. M. (2009). Contagious and spontaneous yawning in autistic and typically developing children. Curr. Psychol. Lett. 25 Available at: http://cpl.revues.org/4810.

[B26] Greco M. Baenninger R. (1989). Self-report as a valid measure of yawning in the laboratory. Bull. Psychon. Soc. 27, 75–76. 10.3758/BF03329903

[B27] Haker H. Kawohl W. Herwig U. Rossler W. (2013). Mirror neuron activity during contagious yawning—an fMRI study. Brain Imaging Behav. 7, 28–34. 10.1007/s11682-012-9189-9 22772979

[B28] Harr A. L. Gilbert V. R. Phillips K. A. (2009). Do dogs (*Canis familiaris*) show contagious yawning? Anim. Cogn. 12, 833–837. 10.1007/s10071-009-0233-0 19452178

[B29] Helt M. S. Eigsti I. M. Snyder P. J. Fein D. A. (2010). Contagious yawning in autistic and typical development. Child Dev. 81, 1620–1631. 10.1111/j.1467-8624.2010.01495.x 20840244

[B30] Hoogenhout M. van der Straaten K. Pileggi L.-A. Malcolm-Smith S. (2013). Young children display contagious yawning when looking at the eyes. J. Child Adolesc. Behav. 1, 2.

[B31] Joly-Mascheroni R. M. Senju A. Shepherd A. J. (2008). Dogs catch human yawns. Biol. Lett. 4, 446–448. 10.1098/rsbl.2008.0333 18682357 PMC2610100

[B32] Kaplan J. T. Freedman J. Iacoboni M. (2007). Us versus them: political attitudes and party affiliation influence neural response to faces of presidential candidates. Neuropsycholgia 45, 55–64. 10.1016/j.neuropsychologia.2006.04.024 16764897

[B33] Keverne E. B. Leonard R. A. Scruton D. M. Young S. K. (1978). Visual monitoring in social groups of Talapoin Monkeys. Anim. Behav. 26, 933–944. 10.1016/0003-3472(78)90157-4

[B34] Liang A. C. Grace J. K. Tompkins E. M. Anderson D. J. (2015). Yawning, acute stressors, and arousal reduction in Nazca booby adults and nestlings. Physiol. Behav. 140, 38–43. 10.1016/j.physbeh.2014.11.029 25498600

[B35] Liuzza M. T. Cazzato V. Vecchione M. Crostella F. Caprara G. V. Aglioti S. M. (2011). Follow my eyes: the gaze of politicians reflexively captures the gaze of ingroup voters. PLoS ONE 6:e25117. 10.1371/journal.pone.0025117 21957479 PMC3177843

[B36] Lount R. B. Jr. Pettit N. C. (2012). The social context of trust: the role of status. Organ. Behav. Hum. Decis. Process. 117, 15–23. 10.1016/j.obhdp.2011.07.005

[B37] Madsen E. A. Persson T. (2012). Contagious yawning in domestic dog puppies (*Canis lupus familiaris*): the effect of ontogeny and emotional closeness on low-level imitation in dogs. Anim. Cogn. 16, 233–240. 10.1007/s10071-012-0568-9 23076724

[B38] Madsen E. A. Persson T. Sayehli S. Lenninger S. Sonesson G. (2013). Chimpanzees show a developmental increase in susceptibility to contagious yawning: a test of the effect of ontogeny and emotional closeness on yawn contagion. PLoS ONE 8:e76266. 10.1371/journal.pone.0076266 24146848 PMC3797813

[B40] Massen J. J. M. Dusch K. Eldakar O. T. Gallup A. C. (2014). A thermal window for yawning in humans: yawning as a brain cooling mechanism. Physiol. Behav. 130, 145–148. 10.1016/j.physbeh.2014.03.032 24721675

[B39] Massen J. J. M. Vermunt D. A. Sterck E. H. M. (2012). Male yawning is more contagious than female yawning among chimpanzees (*Pan troglodytes*). PLoS ONE 7:e40697. 10.1371/journal.pone.0040697 22808234 PMC3394737

[B41] McNelis N. L. Boatright-Horowitz S. L. (1998). Social monitoring in a primate group: the relationship between visual attention and hierarchical ranks. Anim. Behav. 1, 65–69. 10.1007/s100710050008

[B42] Millen A. Anderson J. R. (2010). Neither infants nor toddlers catch yawns from their mothers. Biol. Lett. 7, 440–442. 10.1098/rsbl.2010.0966 21123252 PMC3097853

[B44] Miller M. L. Gallup A. C. Vogel A. R. Vicario S. M. Clark A. B. (2012a). Evidence for contagious behaviors in budgerigars (*Melopsittacus undulatus*): an observational study on yawning and stretching. Behav. Process. 89, 264–270. 10.1016/j.beproc.2011.12.012 22209955

[B43] Miller M. L. Gallup A. C. Vogel A. R. Clark A. B. (2012b). Auditory disturbances promote temporal clustering of yawning and stretching in small groups of budgerigars (*Melopsittacus undulatus*). J. Comp. Psychol. 126, 324–328. 10.1037/a0026520 22268553

[B45] Moyaho A. Rivas-Zamudio X. Ugarte A. Eguibar J. R. Valencia J. (2015). Smell facilitates auditory contagious yawning in stranger rats. Anim. Cogn. 18, 279–290. 10.1007/s10071-014-0798-0 25156806

[B46] Nahab F. B. Hattori N. Saad Z. S. Hallett M. (2009). Contagious yawning and the frontal lobe: an fMRI study. Hum. Brain Mapp. 30, 1744–1751. 10.1002/hbm.20638 18937281 PMC4758456

[B47] Norscia I. Palagi E. (2011). Yawn contagion and empathy in *Homo sapiens*. PLoS ONE 6:e28472. 10.1371/journal.pone.0028472 22163307 PMC3233580

[B48] O’Hara S. J. Reeve A. V. (2011). A test of yawning contagion and emotional connectedness in dogs, *Canis familiaris*. Anim. Behav. 81, 335–340. 10.1016/j.anbehav.2010.11.005

[B49] Overduin-de Vries A. M. Massen J. J. M. Spruijt B. M. Sterck E. H. M. (2012). Sneaky monkeys: an audience effect on rhesus macaque (*Macaca mulatta*) sexual behaviour. Am. J. Primatol. 74, 217–228. 10.1002/ajp.21988 24006540

[B50] Palagi E. Leone A. Mancini G. Ferrari P. F. (2009). Contagious yawning in gelada baboons as a possible expression of empathy. Proc. Natl. Acad. Sci. U.S.A. 106, 19262–19267. 10.1073/pnas.0910891106 19889980 PMC2780782

[B51] Palagi E. Norscia I. Demuru E. (2014). Yawn contagion in humans and bonobos: emotional affinity matters more than species. PeerJ 2:e519. 10.7717/peerj.519 25165630 PMC4137654

[B54] Perner J. Lang B. (1999). Development of theory of mind and executive control. Trends Cogn. Sci. 9, 337–344. 10.1016/S1364-6613(99)01362-5 10461196

[B52] Platek S. M. Critton S. R. Myers T. E. Gallup G. G. (2003). Contagious yawning: the role of self-awareness and mental state attribution. Cogn. Brain Res. 17, 223–227. 10.1016/S0926-6410(03)00109-5 12880893

[B53] Platek S. M. Mohamed F. B. Gallup G. G. Jr. (2005). Contagious yawning and the brain. Cogn. Brain Res. 23, 448–452. 10.1016/j.cogbrainres.2004.11.011 15820652

[B55] Preston S. D. de Waal F. B. M. (2002). Empathy: its ultimate and proximate bases. Behav. Brain Sci. 25, 7–71. 10.1017/S0140525X02000018 12625087

[B56] Provine R. R. (1986). Yawning as a stereotyped action pattern and releasing stimulus. Ethology 72, 109–122. 10.1111/j.1439-0310.1986.tb00611.x

[B57] Provine R. R. (2005). Yawning: the yawn is primal, unstoppable and contagious, revealing the evolutionary and neural basis of empathy and unconscious behavior. Am. Sci. 93, 532–539. 10.1511/2005.56.980

[B58] Romero T. Konno A. Hasegawa T. (2013). Familiarity bias and physiological responses in contagious yawning by dogs support link to empathy. PLoS ONE 8:e71365. 10.1371/journal.pone.0071365 23951146 PMC3737103

[B59] Romero T. Saito A. Hasegawa T. (2014). Social modulation of contagious yawning in wolves. PLoS ONE 9:e105963. 10.1371/journal.pone.0105963 25162677 PMC4146576

[B60] Rundle B. K. Vaughn V. R. Stanford M. S. (2015). Contagious yawning and psychopathy. Pers. Individ. Dif. 86, 33–37. 10.1016/j.paid.2015.05.025

[B61] Schiller F. (2002). Yawning? J. Hist. Neurosci. 11, 392–401. 10.1076/jhin.11.4.392.8540 12557656

[B62] Schurmann M. Hesse M. D. Stephan K. E. Saarela M. Zilles K. Hari R. (2005). Yearning to yawn: the neural basis of contagious yawning. Neuroimage 24, 1260–1264. 10.1016/j.neuroimage.2004.10.022 15670705

[B63] Senju A. Kikuchi Y. Akechi H. Hasegawa T. Tojo Y. Osanai H. (2009). Does eye contact induce contagious yawning in children with autism spectrum disorder. J. Autism Dev. Disord. 39, 1598–1602. 10.1007/s10803-009-0785-5 19533316

[B64] Senju A. Maeda M. Kikuchi Y. Hasegawa T. Tojo Y. Osanai H. (2007). Absence of contagious yawning in children with autism spectrum disorder. Biol. Lett. 3, 706–708. 10.1098/rsbl.2007.0337 17698452 PMC2391210

[B65] Shepherd S. V. Deaner R. O. Platt M. L. (2006). Social status gates social attention in monkeys. Curr. Biol. 16, R119–R120. 10.1016/j.cub.2006.02.013 16488858

[B66] Shoup-Knox M. L. Gallup A. C. Gallup G. G. jr. McNay E. C. (2010). Yawning and stretching predict brain temperature changes in rats: support for the thermoregulatory hypothesis. Front. Evol. Neurosci. 2:108. 10.3389/fnevo.2010.00108 21031034 PMC2965053

[B67] Silva K. Bessa J. de Sousa L. (2012). Auditory contagious yawning in domestic dogs (*Canis familiaris*): first evidence for social modulation. Anim. Cogn. 15, 721–724. 10.1007/s10071-012-0473-2 22526686

[B68] Trautmann-Lengsfeld S. A. Domínguez-Borràs J. Escera C. Herrmann M. Fehr T. (2013). the perception of dynamic and static facial expressions of happiness and disgust investigated by ERPs and fMRI constrained source analysis. PLoS ONE 8:e66997. 10.1371/journal.pone.0066997 23818974 PMC3688578

[B69] Usui S. Senju A. Kikuchi Y. Akechi H. Tojo Y. Osanai H. (2013). Presence of contagious yawning in children with autism spectrum disorder. Autism Res. Treat. 2013, 971686. 10.1155/2013/971686 23970970 PMC3736493

[B70] Yoon J. M. D. Tennie C. (2010). Contagious yawning: a reflection of empathy, mimicry, or contagion? Anim. Behav. 79, e1–e3. 10.1016/j.anbehav.2010.02.011

